# Dextran-Curcumin Nanoparticles as a Methotrexate Delivery Vehicle: A Step Forward in Breast Cancer Combination Therapy

**DOI:** 10.3390/ph13010002

**Published:** 2019-12-25

**Authors:** Manuela Curcio, Giuseppe Cirillo, Paola Tucci, Annafranca Farfalla, Emilia Bevacqua, Orazio Vittorio, Francesca Iemma, Fiore Pasquale Nicoletta

**Affiliations:** 1 Department of Pharmacy, Health and Nutritional Sciences, University of Calabria, 87036 Rende (CS), Italy; manuela.curcio@unical.it (M.C.); paola.tucci@unical.it (P.T.); annafranca.farfalla@gmail.com (A.F.); emiliabevacqua@libero.it (E.B.); francesca.iemma@unical.it (F.I.); fiore.nicoletta@unical.it (F.P.N.); 2 Children’s Cancer Institute, Lowy Cancer Research Centre, UNSW, Sydney, NSW 2031, Australia; OVittorio@ccia.org.au; 3 ARC Centre of Excellence for Convergent BioNano Science and Technology, Australian Centre for NanoMedicine, UNSW, Sydney, NSW 2052, Australia; 4 School of Women’s and Children’s Health, Faculty of Medicine, UNSW, Sydney, NSW 2052, Australia

**Keywords:** polyphenol conjugate, self-assembling nanoparticles, curcumin, breast cancer, synergism

## Abstract

With the aim to effectively deliver methotrexate (MTX) to breast cancer cells, we designed a nanocarrier system (DC) derived from the self-assembly of a dextran-curcumin conjugate prepared via enzyme chemistry with immobilized laccase acting as a solid biocatalyst. Nanoparticles consisted of homogeneously dispersed nanospheres with a mean diameter of 290 nm, as characterized by combined transmission electron microscopy and dynamic light scattering investigations. DC was able to control the MTX release overtime (t_1/2_ value of 310 min), with cell internalization studies proving its presence inside MCF-7 cytoplasm. Finally, improved MTX efficacy was obtained in viability assays, and attributed to the synergy of curcumin moieties and loaded MTX as underlined by a combination index (CI) < 1.

## 1. Introduction

Cancer is still one of the leading causes of death worldwide, resulting in a continuous increase in the economic and financial burden for cancer treatment and research [1,2]. Surgery and chemotherapy remain the first line cancer treatments, with chemotherapeutic protocols often consisting in a combination of two or more bioactive agents, each used at lower dosage [3,4,5]. The advantages of such approach is the possibility to simultaneously target different cellular pathways [6,7], thus increasing the therapeutic efficiency, reducing the toxic side effects, and hindering the insurgence of multi-drug resistance [8]. Moreover, with the aim to improve the therapeutic performance, great efforts have been made in both the fabrication of targeted nanocarriers [9] and the exploration of alternative anticancer agents [10,11]. To this regard, polyphenols have been shown to possess strong anticancer activity in vitro, although they suffering from some pharmacokinetic drawbacks, e.g., inefficient systemic delivery, poor bioavailability, and high required dosage, significantly hindering their use in the clinic [12]. The fabrication of polyphenol nanoformulations, possessing high surface area to volume ratio and tunable physicochemical properties, was proposed as a promising strategy to address these matters, allowing a “bench to bedside” translation for human use to be hypothesized [13]. Among the wide number of approaches reported in the literature [14], the use of polymeric materials for the synthesis of either polyphenol delivery vehicles or bioconjugates, has been proved to be effective for their stabilization and for the improvement of their bioavailability [15]. The latter approach offers the further advantage of conferring an added value to the polymeric counterpart, which can be used for the preparation of a functional drug delivery system, in which the biological effect is related to both the loaded drug and the carrier itself [16].

We chose curcumin (CUR) as polyphenol moiety because of its well-known biological properties and ability to be used in combination chemotherapy protocols for the treatment of a wide number of cancer diseases with high efficiency [17,18]. In the literature, different CUR-polymer conjugates (e.g., xylan [19], hyaluronic acid [20], chitosan [21], Dextran [22], PEG [23], and albumin [24]) were reported with the purpose to improve the anticancer performance of the polyphenol and to synergize conventional chemotherapeutics such as methotrexate (MTX), doxorubicin, and paclitaxel. Herein, we propose the enzymatic conjugation of CUR to Dextran (DEX-CUR), a glucose homopolysaccharide showing high biocompatibility and chemical versatility [25], in order to transfer the biological activity of CUR to a macromolecular systems able to self-assembly (DC), encapsulate, and control the release of MTX. Finally, biological assays were performed to check the ability of the conjugate to be internalized within breast cancer cells and synergize the cytotoxic effect of MTX. 

## 2. Results

### 2.1. Synthesis and Characterization of Nanoparticles

**Scheme 1:**
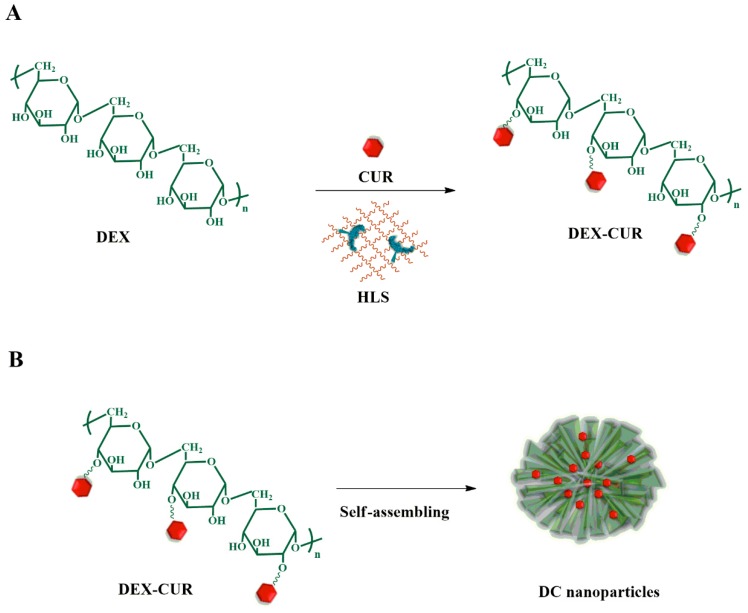
Schematic representation of the fabrication of DC nanoparticles. (**A**) Synthesis of DEX-CUR conjugate by immobilized laccase (HLS); (**B**) Conjugate self-assembly.

**Figure 1:**
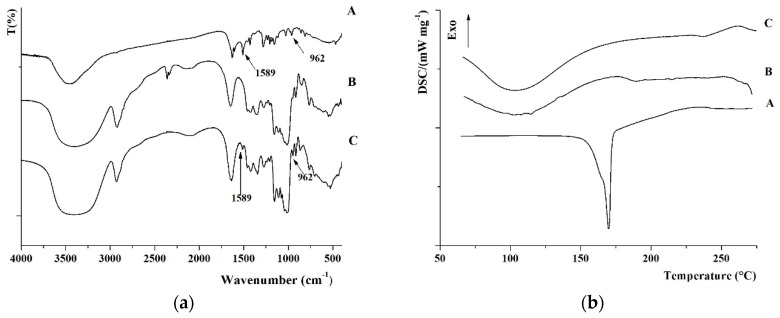
(**a**) FT-IR spectra and (**b**) DSC thermograms of CUR (A), DEX (B), and DEX-CUR (C).

DC nanoparticles were prepared by a two-steps procedure, namely (i) the synthesis of DEX-CUR and (ii) the self-assembly of the conjugate in water media (Scheme 1):

For the synthesis of the DEX-CUR conjugate, laccase immobilized into acrylate hydrogel film was used as solid biocatalyst in a fully green procedure [26]. FT-IR and DSC analyses unequivocally proved the effective conjugation of the polyphenol to the polysaccharides backbone. In detail, the FT-IR of the DEX-CUR conjugate showed the appearance of the CUR signature peaks at 1589 (C-C aromatic stretching) and 968 (enolic bending) cm^−1^ (Figure 1a) [27], whereas no transition related to the melting point of the polyphenol (169 °C) was detected in the DSC thermogram (Figure 1b), as a consequence of the loss of its crystalline structure upon conjugation [28].

In order to determine the degree of functionalization, available phenolic groups were determined by means of a Folin-Ciocalteu assay [29], obtaining a value of 23.9 mg CUR per gram of conjugate. Moreover, the absence of significant differences (*p* > 0.05) between the Trolox equivalent antioxidant capacity (TEAC) [30] of free (2.43) and conjugated (2.23) CUR confirmed that the conjugation did not interfere with the CUR scavenging activity.

**Figure 2:**
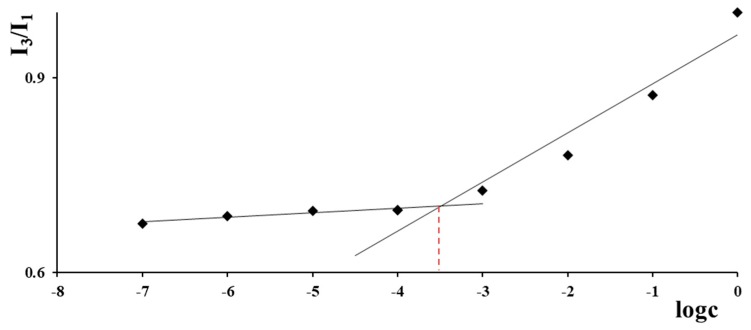
Dependence of the pyrene fluorescence spectrum signals on DC concentration in PBS 0.01 mol L^−1^, pH 7.4.

The insertion of CUR in the DEX backbone was expected to modify the hydrophobic to hydrophilic balance within the polymeric structure, conferring self-assembling properties to the final conjugates [31]. The CAC, estimated from the dependence of pyrene fluorescence spectra (I_384_/I_373_ ratio) on the logarithm of conjugate concentration (Figure 2) is an important parameter for the evaluation of the stability of the self-assembling nanostructures upon dilution.

In the assay, the I_384_/I_373_ ratio remained almost unchanged at low concentrations, whereas a sharp change of the intensity was observed with an increased amount of conjugate, indicating the onset of self-assembly. From the crossover point, a CAC of 0.31 µg mL^−1^ was calculated, suggesting the suitability of the proposed nanoparticles for systemic administration due to its stability under high dilution conditions [32].

**Figure 3:**
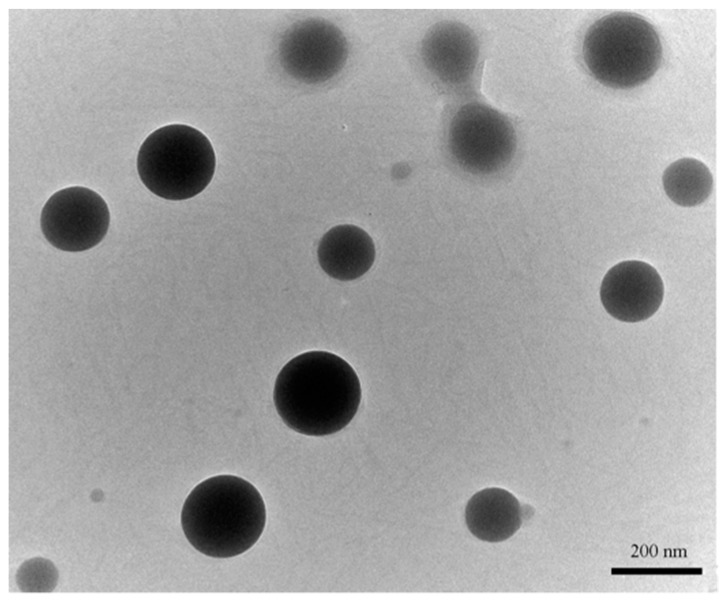
TEM images of DC, showing microspheres with an average diameter of 290 nm.

DC nanoparticles were easily prepared by dispersion of DEX-CUR conjugate in PBS (0.01 mol L^−1^, pH 7.4) in a final concentration of 0.5 mg mL^−1^, and characterized by TEM and DLS analyses to determine shape, size, and polydispersity. As a result, spherical nanoparticles with mean diameter of 290 nm (Figure 3) and a PDI of 0.21 were observed.

### 2.2. Drug Release Experiments

**Figure 4:**
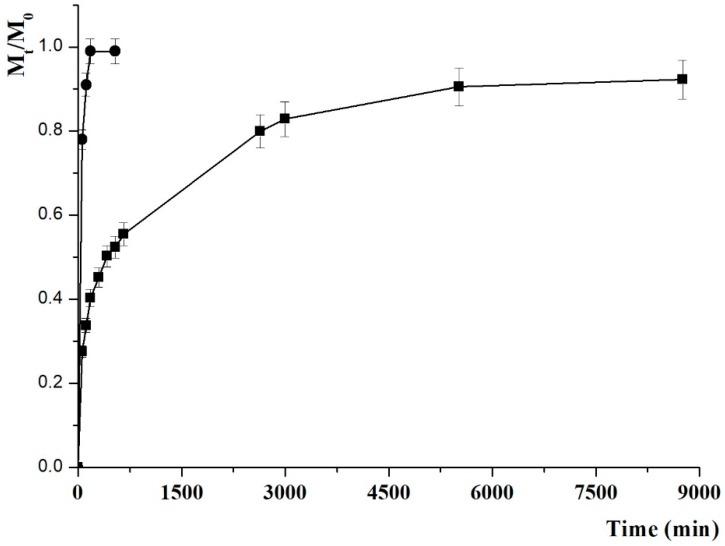
Release profiles of free (●) and DC-loaded (■) MTX in PBS 0.01 mol L^−1^, pH 7.4.

MTX-loaded DC (MTX@DC) were obtained by using a MTX solution in PBS (0.01 mol L^−1^, pH 7.4) as dispersing medium for the DC conjugate and selecting a drug to carrier ratio of 20% (by weight). The MTX release profile in the same above-mentioned medium, recorded by a dialysis method, is depicted in Figure 4. Unloaded MTX showed a fast diffusion in the releasing media (M_t_/M_0_ of 90% after 2h), while a more sustained release (M_t_/M_0_ of 34% at the same experimental time) was recorded when the drug is loaded into the nanoparticle system as a consequence of the partition of the drug between the carrier and solvent phases.

For a better analysis, the release of MTX was analyzed by applying a mathematical model describing such partition phenomenon [33], with the physicochemical affinity of the solute between the carrier and the release medium expressed by the α parameter according to Equation (1):

(1) $$\alpha \;=\;\frac{F_{max}}{1-F_{max}}$$

where *F_max_* is the maximum value of relative release (M_t_/M_0_) and was found to be of 92% after 92 h.

In this model, the release can follow a reversible first- or second-order kinetic, according to the following Equations (2) and (3), respectively:

(2) $$\frac{M_{t}}{M_{0}}=F_{max}\left(1-e^{-\left(\frac{k_{R}}{F_{max}}\right)t}\right)$$

(3) $$\frac{M_{t}}{M_{0}}=\frac{F_{max}\left(e^{2\left(\frac{k_{R}}{\alpha }\right)t}-1\right)}{1-2F_{max}+e^{2\left(\frac{k_{R}}{\alpha }\right)t}}$$

where *k_R_* is the release rate constant.

Higher *R*^2^ values were recorded (0.9359 vs. 0.8866) when Equation (3) was applied, allowing a reversible second-order kinetics to be assumed (Table 1). More information can be obtained by considering the *k_R_* (2.03 × 10^−3^) and the (t_1/2_ = 5.1 h) values, with the latter being expressed as the time required for the drug to reach 50% of F_max_ (Equation (4)):

(4) $$t_{1/2}=\frac{\alpha }{2kR}\ln\left(3-2F_{max}\right)$$

The results clearly proved the ability of DC to control the MTX release over time, and are consistent with literature data evaluating the drug delivery performance of nanoparticle systems [34,35].

### 2.3. Biological Characterization

DC nanoparticles are expected to improve the therapeutic efficacy of the payload by virtue of two main factors: (i) a relatively slow release allowing the retention of the drug until it reaches the site of action; (ii) the synergistic anticancer activity of CUR in combination with MTX. To prove these statements, viability experiments were performed using MCF-7 breast cancer cells as in vitro model. At first, unconjugated DEX was found to not significantly affect cell viability in all the tested concentrations (*p* > 0.05, data not shown), according to literature data reporting no toxicity of carrier systems based on this polysaccharide material [36,37].

**Figure 5:**
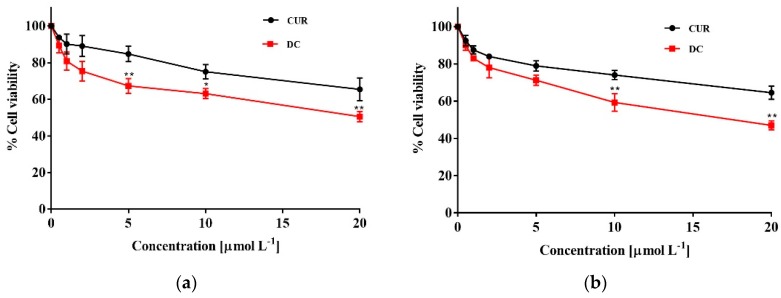
MCF-7 cells treated with increasing concentrations of CUR and DC for (**a**) 24 h and (**b**) 48 h. * *p* < 0.01, ** *p* < 0.001 vs. free CUR at equivalent concentration.

Then, the effect of conjugation on CUR anticancer activity was assessed by comparing the cell viability after 24 and 48 h treatment with CUR and DC at 0–20 µmol L^−1^ CUR equivalent concentrations. The experimental results, represented in Figure 5, demonstrated that the biological activity of CUR was retained and even significantly improved upon conjugation. After 48 h incubation with 20 µmol L^−1^ DC, indeed, cell viability was reduced up to 47% (65% in the case of free CUR). The IC_50_ values of DC (expressed as CUR equivalent concentration) roughly reduced to half of the reference CUR (from 33 to 18.8 μmol L^−1^ and from 31.7 to 16.9 μmol L^−1^ at 24 and 48 h, respectively), allowing a favorable interaction between carrier and cytotoxic drug to be hypothesized. 

The improvement of CUR biological activity can be ascribed to the fast internalization of Dextran into the cancer cells [38]. It was previously proved, indeed, that fluorescein isothiocyanate-Dextran can be observed inside cells after 20 min of incubation, suggesting its ability to promote a highly efficient CUR uptake by MCF-7 cells. 

**Figure 6:**
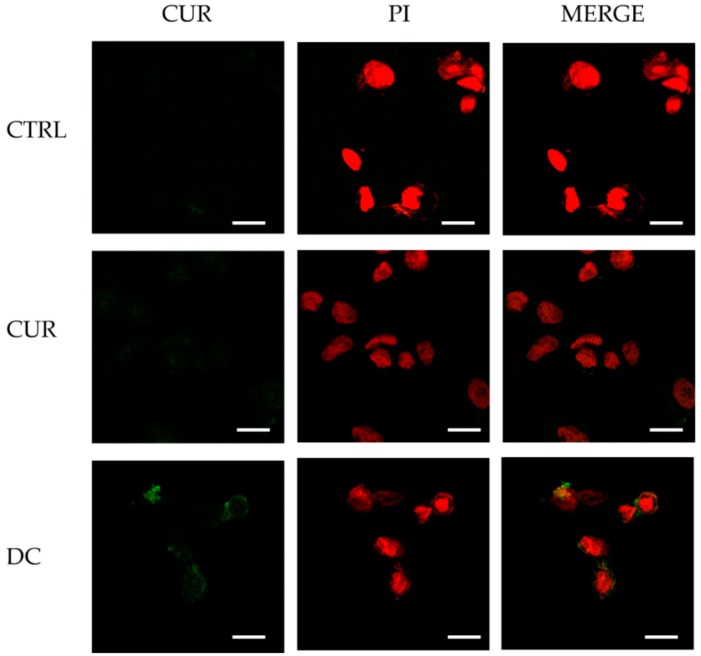
Confocal images of MCF-7 cells exposed to vehicle alone (CTRL) or treated with 20 µmol L^−1^ of curcumin (CUR) or 20 µmol L^−1^ of Dextran/curcumin (DEX/CUR) for 4 h. Scale bar: 20 µm.

The rapid internalization of DC nanoformulation was confirmed by confocal microscopy (Figure 6), where the presence of green fluorescence signal of CUR was detected in the cytoplasm. Moreover, by the quantification of the fluorescence intensity, an enhanced cell uptake was demonstrated for DC treatments (*p* < 0.05, per t-test).

**Figure 7:**
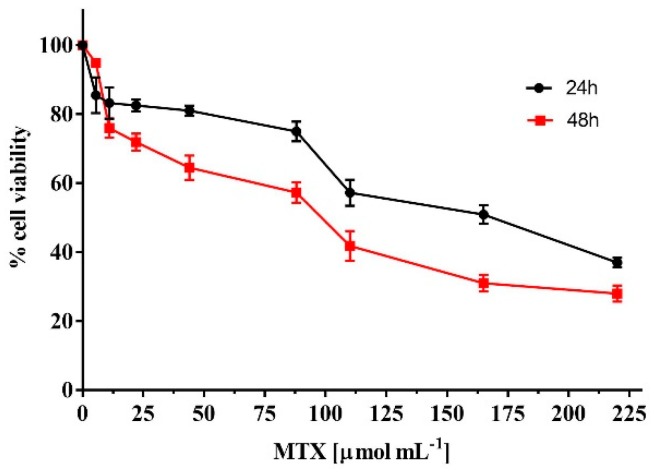
MCF-7 cells treated with increasing concentrations of methotrexate (MTX) for 24 or 48 h.

**Figure 8:**
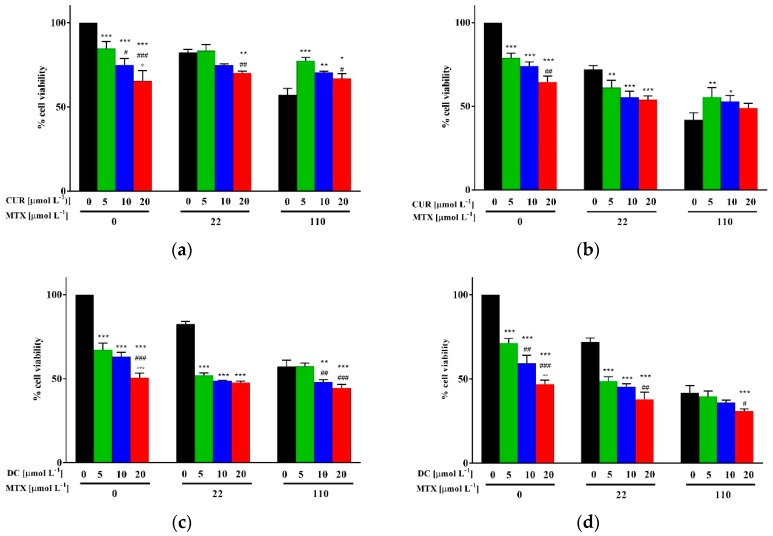
MCF-7 cells treated with different concentrations of (**a**,**b**) CUR and (**c**,**d**) DC in combination with MTX 22 and 110 µmol mL^−1^ for (**a**,**c**) 24 or (**b**,**d**) 48 h. Within each MTX treatment, * *p* < 0.01, ** *p* < 0.001, *** *p* < 0.0001 vs. [CUR or DC] 0 µmol mL^−1^; ^#^ *p* < 0.01, ^##^ *p* < 0.001, ^###^ *p* < 0.0001 vs. [CUR or DC] 5 µmol mL^−1^; ° *p* < 0.01, °° *p* < 0.001, °°° *p* < 0.0001 vs. [CUR or DC] 10 µmol mL^−1^.

Subsequently, after assessing the typical dose-dependent toxicity profile of free MTX (Figure 7), viability experiments were performed with MTX@DC samples at different drug to carrier ratios (Figure 8b).

In control experiments, viability data obtained employing CUR/MTX combinations at the same ratios were recorded (Figure 8a). 

The results proved that cell viability was reduced up to 47% by treatments with 22 µmol L^−1^ MTX and 5 µmol L^−1^ DC, suggesting the possibility to significantly reduce the dose of MTX for an effective anticancer treatment. To better analyze the data, and to highlight the dose-effect data of combined vs. single drug treatments Combination Indexes (CI, Table 1) were determined by employing the Chou-Talalay method (Equation (5)) [39]:

(5) $$CI=\frac{D_{1}}{D_{x1}}+\frac{D_{2}}{D_{x2}}$$

where *D*_1_ and *D*_2_ are the doses of drug 1 (CUR or DC) and drug 2 (MTX) in the combination, while *D_x_*_1_ and *D_x_*_2_ are the doses of drug 1 and drug 2 that would give the same effect as that of the combination (*D*_1_ + *D*_2_). The doses *D_x_*_1_ and *D_x_*_2_ were estimated from the dose-effect data of single drug treatments. 

According to this theorem, CI < 1 indicates synergism; CI = 1 additive effect, and CI > 1 antagonism. Compared to free MTX/CUR combinations, MTX@DC samples were thus found to be more effective in reducing cell viability at both 24 and 48 h, exerting nearly additive or synergistic effects at MTX concentration of 110 and 22 μmol L^−1^, respectively (CI values from 0.44 to 1.1). On the other hand, when the cancer cells were treated with free drugs for 24h, antagonistic effects were observed for all the tested concentrations (CI > 1.2), becoming synergistic only after 48 h incubation with 22 μmol L^−1^ MTX. This is in accordance with our expectation and with literature data proving the ability of polyphenol conjugates and nanoparticle systems to increase the therapeutic efficiency of conventional anticancer drugs [16,40]. The therapeutic performance of the proposed nanoformulation, which can potentially accumulate in cancer tissues by the enhanced permeability and retention (EPR) effect, will be further investigated in suitable xenograft models, with the aim to evaluate both the pharmacokinetics profiles and the anticancer activity in vivo. 

## 3. Materials and Methods 

### 3.1. Synthesis of Curcumin Conjugate

The synthetic procedure adopted for the synthesis of DEX–CUR conjugate was as follows: 300 mg DEX (Mr 40,000) was dissolved in 20 mL PBS (10^3^ mol L^−1^, pH 6.8)/DMSO mixture (25/75 vol/vol) and then 36 mg CUR and immobilized laccase (0.12 U), prepared as previously reported [26] were added and allowed to react at 37 °C at 70 rpm for 12 h. Then, the DEX–CUR conjugate was dialyzed (dialysis tubes of 6–27/3200 Medicell International Ltd (Liverpool, UK), MWCO: 12,000–14,000 Da) against (i) reaction medium and (ii) distilled water to remove the unreacted CUR and the residual DMSO, respectively. Finally, freeze-drying procedure (Micro Modulyo, Edwards Lifesciences, Irvine, CA, USA) allowed to recover a vaporous solid. High pressure liquid chromatography (HPLC) analysis of the dialysis medium was performed to check the complete removal of unreacted CUR. The HPLC system consisted of a Jasco PU-2089 Plus liquid chromatograph equipped with a 7725i injector (Rheodyne, Rohnert, CA, USA), fitted with a 20 mL loop, a Jasco UV-2075 HPLC detector operating at 420 nm, and a Jasco-Borwin integrator (Jasco Europe s.r.l., Milan, Italy). The stationary phas was a Tracer Excel 120 ODS-A column, particle size 5 mm, 15 × 0.4 cm (Barcelona, Spain) [24]. All chemicals were obtained from Merck KGaA (Darmstadt, Germany).

### 3.2. Characterization of Conjugate

FT-IR spectra were recorded as pellets in KBr in the range 4000–400 cm^−1^ using a Jasco FT-IR 4200 spectrophotometer (Jasco Europe s.r.l.) with resolution 1 cm^−1^.

Calorimetric analysis was carried out using a DSC200 PC differential scanning calorimeter (Netzsch, Selb, Germany). In a standard procedure about 1.0 mg dried sample was placed in an aluminum pan, and then sealed tightly by an aluminum lid. The thermal analyses were performed from 60 to 300 °C under a dry nitrogen atmosphere with a flow rate of 25 mL min^−1^ and heating rate of 5 °C min^−1^.

Total available phenolic groups, calculated by the Folin-Ciocalteu procedure [29], were expressed as CUR equivalent (mg) per gram of polymer by using the equation obtained from the calibration curve of the free CUR. Briefly, 50 mg amount DEX–CUR conjugate was dissolved in 6.0 mL distilled water and then 1.0 mL Folin–Ciocalteu reagent was added. After 3 min stirring, 3 mL Na_2_CO_3_ (7.5%) was added and the mixture allowed to stand for 2 h with intermittent shaking. The absorbance was measured at 760 nm against blank DEX solution prepared in the same condition on an Evolution 201 spectrophotometer (ThermoFisher Scientific, Hillsboro, OR, USA).

For the determination of the Trolox equivalent antioxidant capacity (TEAC) DEX–CUR (0.076 mg mL^−1^) was added to 2,2′-azino-bis(3-ethylbenzothiazoline-6-sulfonic acid (ABTS) in the concentration range 0–1.23 × 10^−4^ mol L^−1^ and, after 6 min incubation at 37 °C, the absorbance was recorded at 734 nm on an Evolution 201 spectrophotometer (ThermoFisher Scientific) [30]. *TEAC* was calculated by the following Equation (6):

(6) $$TEAC=\frac{C}{1.9\cdot \left[CUR\right]}$$

where 1.9 represents the amount of ABTS (mol) scavenged per mol of Trolox, and [*CUR*] is the *CUR* equivalent concentration (mol L^−1^) in the sample. The maximal amount of ABTS scavenged by the tested concentration of the antioxidant (*C*) was calculated by plotting the reduction of ABTS concentration against its initial concentration (Equation (7)):

(7) $$y=C\left(1-e^{-bx}\right)$$

where *x* and *y* are the initial ABTS concentration and the reduction in ABTS concentration, respectively.

### 3.3. Preparation of Nanoparticles and Methotrexate Loading

Un-loaded (DC) and MTX loaded (MTX@DC) nanoparticle systems were prepared by a method previously described [41,42]. Briefly, 2.5 mg DEX–CUR conjugate were added to 5.0 mL PBS solution (10^−3^ M, pH 7.4), or MTX solution (0.5 mg mL^−1^) in PBS under stirring at room temperature for 3 h. 

Morphological analysis of nanoparticles was carried out using transmission electron microscopy (TEM, Tecnai F30, FEI, Hillsboro, OR, USA). A drop of the nanoparticles dispersion was placed on a Cu TEM grid (200 mesh, Plano GmbH, Wetzlar, Germany), and the sample in excess was removed using a piece of filter paper. A drop of 2% (w/v) phosphotungstic acid solution was then deposited on the carbon grid and left to stay for 2 min. Once the excess of staining agent was removed, the samples were air-dried and the thin film of stained nanoparticles was observed.

Size distributions were determined using a 90 Plus Particle Size Analyzer DLS equipment (Brookhaven Instruments Corporation, New York, NY, USA) at 25 °C. The autocorrelation function was measured at 90° and the laser beam operated at 658 nm. The polydispersity index (PDI) was directly obtained from the instrumental data fitting procedures by the inverse Laplace transformation and Contin methods. PDI values ≤ 0.3 indicate homogenous and mono-disperse populations [43].

### 3.4. In Vitro Drug Release

The release experiments were performed in phosphate buffered saline (10^−3^ mol L^−1^) at pH 7.4 by inserting MTX@DC in PBS (1.5 mL) into a dialysis bag (MWCO: 12,000–14,000 Da), and dialyzed against fresh PBS (13.5 mL). At suitable time intervals, the amount of MTX in the release media was determined by HPLC analysis. The stationary and mobile phases consisted of a Tracer Excel 120 ODS-A column, particle size 5 mm, 15 × 0.4, and a 0.05% w/w H_3_PO_4_ aqueous solution/methanol (77/23, vol/vol) with a flow rate of 1.0 mL min^−1^, respectively.

### 3.5. Cell Culture

The human breast cancer MCF-7 cell line used was obtained from American Type Culture Collection (ATCC, In Vitro Technologies Pty. Ltd., Melbourne, Victoria, Australia) and maintained in DMEM supplemented with 10% fetal bovine serum, 1% glutamine and 1% penicillin/streptomycin at 37 °C and 5% CO_2_. All chemicals were from Invitrogen (ThermoFisher Scientific, Waltham, MA, USA).

### 3.6. Viability Assay

MCF-7 cells were seeded at a density of 5 × 10^4^ cells/well (0.5 mL/well) in 24-well plates 24 h before to be treated. Cell were then incubated with different concentrations of CUR or DC (0.5, 1, 2, 5, 10 and 20 μmol L^−1^], and/or MTX (22 or 110 μmol L^−1^) for 24 or 48 h. To measure cell viability, the (3-(4,5-dimethylthiazol-2-yl)-5-(3-carboxymethoxyphenyl)-2-(4-sulfophenyl)-2*H*-tetrazolium) (MTT) solution (0.5 mg mL^−1^) was added to each well and the cells were incubated in the dark for an additional 4 h. Then, 200 μL of DMSO were added to each well and the plates were agitated for 10 min at room temperature. The absorbance of the samples was measured at 570 nm using a microplate reader (BioTek Instruments, Winooski, VT, USA). The optical density (OD) was calculated as the difference between the absorbance at the reference wavelength and that at the test wavelength. Percent viability was calculated as (OD of treated sample/control OD) × 100. 

### 3.7. Staining for Confocal Microscopy

Using the intrinsic fluorescence property of CUR, the cellular uptake of the drug was visualized in MCF-7 cells using a Fluoview FV300 confocal laser scanning microscope (Olympus Corporation, Tokyo, Japan). To this end, the cells were grown overnight on coverslips, washed in PBS and incubated with vehicle alone or with 20 μM of CUR or DC for 4 h at 37 °C. After three washes in PBS, cells were fixed in 4% paraformaldehyde for 15 min at room temperature and permeabilized in 0.5% Triton X-100 in PBS supplemented with 3% bovine serum albumin for 10 min at RT. Cells were next incubated with RNasi 100 μg mL^−1^ for 20 min at 37 °C followed by incubation with 500 nM of propidium iodide (PI) for 5 min at RT to selectively label DNA. Finally, cells were washed three times with PBS and the coverslips mounted with Aquapolymount antifading solution (Polysciences, Hirschberg an der Bergstrasse, Germany) on glass slides and observed under a confocal microscope. All chemicals were from Invitrogen (ThermoFisher Scientific).

### 3.8. Statistical Analysis

Data are reported as mean values ± SD of at least three independent experiments. Unpaired Student t-test or one-way analysis of variance (ANOVA) followed by Dunnett’s method were used to generate statistical analysis, using the GraphPad Prism program (GraphPad Software, San Diego, CA, USA). *p* values < 0.01 were considered statistically significant. 

## 4. Conclusions

In this work, we provide experimental evidence that a novel self-assembing DEX-CUR conjugate is a capable delivery vehicle for the release of cytotoxic agents such as MTX acting synergistically to increase the therapeutic effects on MCF-7 cancer cells. The synthetic strategy involved the preparation of DEX-CUR prepared via oxidative coupling reaction catalyzed by immobilized Laccase, and then its self-assembly to form the final nanoparticle systems. The physico-chemical characterization allowed determination of morphological features of the bioconjugate. The MTX release profile was found to be extended overtime, as a consequence of the encapsulation of the drug within the carrier structure, whereas the in vitro viability assays clearly showed the enhancement of cytotoxic activity due to both the presence of CUR moieties within the polysaccharide backbone and the fast internalization of the nanoparticles by MCF-7. The obtained experimental results makes the proposed nano-formulation a promising carrier for cancer treatment.

## Figures and Tables

**Table 1:** Combination index of MTX/CUR (or DC) combinations.

MTX (µM)	CUR Equivalent Concentration (µM)											
	24 h						48 h					
	Free CUR			DC			Free CUR			DC		
	5	10	20	5	10	20	5	10	20	5	10	20
**22**	1.59	1.23	1.61	0.44	0.65	1.11	0.59	0.66	0.98	0.50	0.70	0.96
**110**	2.91	2.27	2.46	1.13	1.11	1.15	1.60	1.63	1.72	1.03	1.10	1.17
